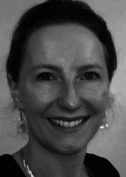# Optical Biosensors for Virus Detection: Prospects for SARS‐CoV‐2/COVID‐19

**DOI:** 10.1002/cbic.202000744

**Published:** 2020-12-09

**Authors:** Hemanth Maddali, Catherine E. Miles, Joachim Kohn, Deirdre M. O'Carroll

**Affiliations:** ^1^ Department of Chemistry and Chemical Biology Rutgers University 123 Bevier Road Piscataway NJ 08854 USA; ^2^ Department of Materials Science and Engineering Rutgers University 607 Taylor Road Piscataway NJ 08854 USA

**Keywords:** colorimetry, COVID-19, fluorescence, optical biosensors, plasmons, virus detection

## Abstract

The recent pandemic of the novel coronavirus disease 2019 (COVID‐19) has caused huge worldwide disruption due to the lack of available testing locations and equipment. The use of optical techniques for viral detection has flourished in the past 15 years, providing more reliable, inexpensive, and accurate detection methods. In the current minireview, optical phenomena including fluorescence, surface plasmons, surface‐enhanced Raman scattering (SERS), and colorimetry are discussed in the context of detecting virus pathogens. The sensitivity of a viral detection method can be dramatically improved by using materials that exhibit surface plasmons or SERS, but often this requires advanced instrumentation for detection. Although fluorescence and colorimetry lack high sensitivity, they show promise as point‐of‐care diagnostics because of their relatively less complicated instrumentation, ease of use, lower costs, and the fact that they do not require nucleic acid amplification. The advantages and disadvantages of each optical detection method are presented, and prospects for applying optical biosensors in COVID‐19 detection are discussed.

## Graphical abstract

**Fast facts at the frontline**: Optical biosensors have led to significant advancements in virus detection and imaging capabilities. Coupled with advanced instrumentation, they have enabled higher sensitivities while increasing the rate at which samples can be tested. These techniques can be developed into point‐of‐care (POC) diagnostics for viral detection and are promising alternatives to detect COVID‐19.

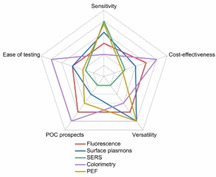

## Introduction

**Figure 1:**
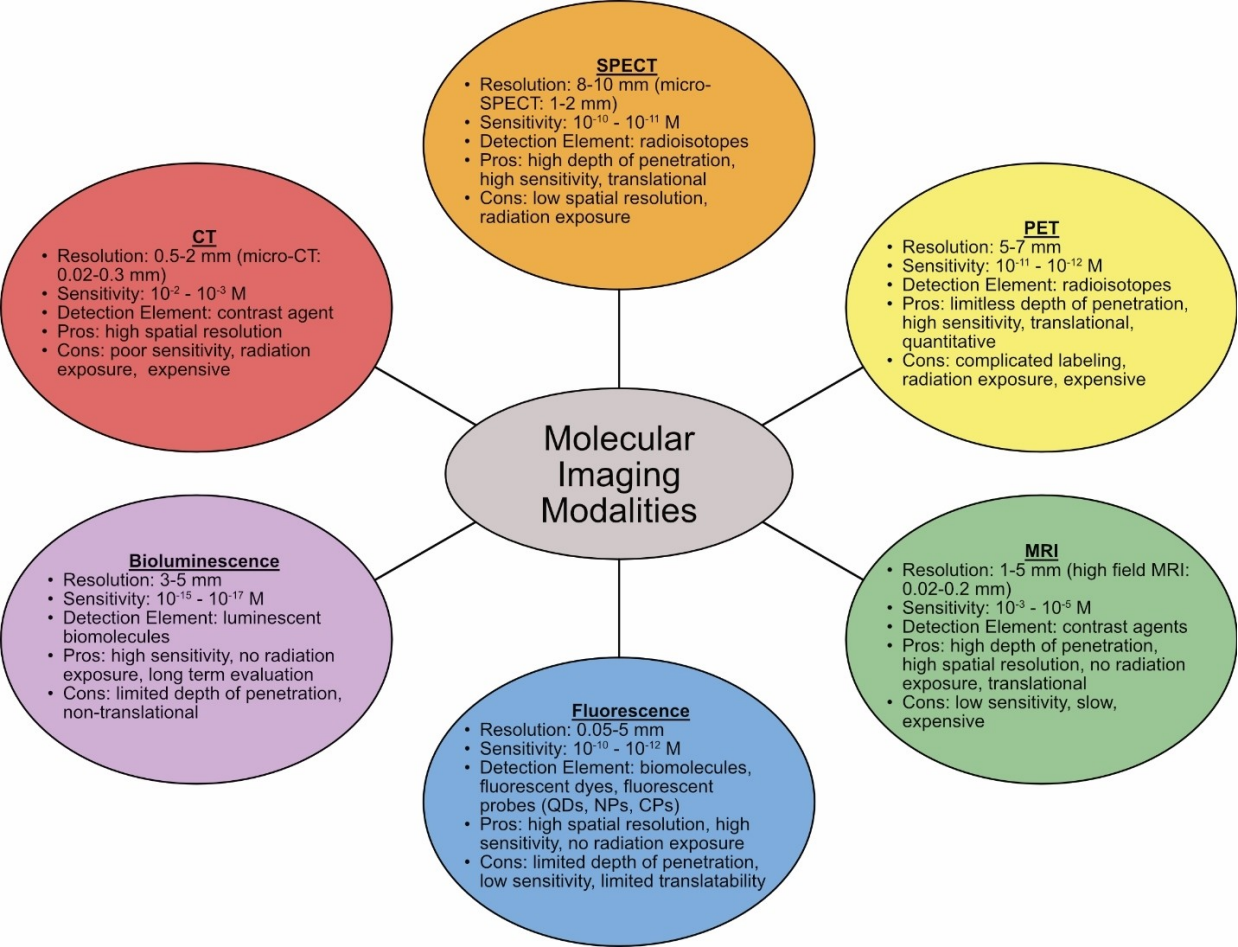
Comparison of CT, SPECT, PET, MRI, fluorescence, and bioluminescence molecular‐imaging modalities as related to resolution, sensitivity, detection element, pros and cons.^[13]^

The novel coronavirus disease 2019 (COVID‐19) has caused an unprecedented surge in virus research, in particular improving testing and diagnostics. The rapid global spread of COVID‐19, caused by the severe acute respiratory syndrome coronavirus 2 (SARS‐CoV‐2) and slow and sometimes inaccurate testing has highlighted the need for more advanced imaging and detection techniques. The current standards for virus imaging include computed tomography (CT), single photon emission computed tomography (SPECT), and positron emission tomography (PET).^[1]^ These methods are costly, have low resolution, and in the case of CT can only detect signs of virus infection (i. e., pneumonia or lung lesions),^[2]^ although recently CT has been used as an additional technique for COVID‐19 diagnosis.^[3]^ Often enzyme‐linked immunosorbent assay (ELISA) or reverse‐transcription polymerase chain reaction (RT‐PCR) are linked with immunofluorescence to detect pathogens and viruses.^[4]^ Currently, RT‐PCR is the gold standard for SARS‐CoV‐2 detection; however, this is a multi‐step technique which involves purification, nucleic acid amplification, and fluorescence detection.^[5]^ The process is laborious, requires a trained operator, can report a number of false‐negatives, and has limited availability in resource‐limited settings.^[6]^ A comparison between different molecular imaging modalities is shown in Figure 1.

Optical biosensors present an alternative method for virus detection due to their safe, straight‐forward use, and cost‐effective technology, including eliminating the need for nucleic acid amplification.^[7]^ Fluorescence, surface plasmons, and colorimetry techniques have all been used previously for the detection of HIV, Ebola, norovirus, and influenza virus, amongst others.^[8]^ These techniques have been used in nano‐biosensors to allow for targeted virus detection and single virus imaging.^[9]^ Optical biosensors can also be used as point‐of‐care (POC) diagnostic tools. POC diagnostics use collected samples without the need for sample preparation, require low costs of test manufacturing, and do not require trained personal or expensive analysis equipment.^[10]^ To the best of our knowledge, only a handful of optical biosensors are currently on the market for virus detection, most comprising of lab‐on‐a‐chip (LOC) techniques that amplify nucleic acids for fluorescent analysis.^[11]^ Improving optical imaging, in particular single virus imaging, has the potential to be used to track and monitor virus replication, cell interaction, and termination in order to more quickly and efficiently develop treatment options. Ongoing research to apply these imaging techniques to detect COVID‐19 has already begun;^[12]^ however, further work is necessary to bring these technologies from benchtop to market. This minireview examines multiple optical techniques and their applications in virus detection and presents a perspective on their potential use to detect SARS‐CoV‐2.

## Methods of Optical Biosensing

Optical biosensing can combine detection and imaging which can provide a deeper understanding of a pathogen in addition to detecting it in biological samples.^[14]^ Optical bioimaging combines advanced optical methods with pathogen‐specific tracers, allowing for targeting and detection of abnormalities in a disease pathway at the molecular stage. Some of the advantages of optical bioimaging over conventional imaging methods like MRI, CT and PET include femtomolar sensitivity, high spatial resolution, non‐invasive and non‐ionizing imaging, low equipment/personnel cost, ease of mobilization, quantitative results and short processing time.[^7^, ^15^] While useful for *ex vivo* diagnostics of processed biological samples, direct imaging without sample preparation has many challenges: the presence of dense tissues in biological samples introduces loss of light directionality that results in a higher degree of scattering.^[16]^ The dense biological tissues have high absorbance that reduces the light intensity, resulting in a subsequent decrease in the signal‐to‐noise ratio. The scattering and absorbing effects of these biological samples can be overcome by employing excitation and emission wavelengths in the near‐infrared window (NIR 1) (700–900 nm)^[17]^ or a second NIR window (NIR 2; 1.0–1.7 μm).^[18]^ Recent research involving fluorescence, surface plasmon and surface enhanced Raman scattering (SERS)‐based detection and imaging has advanced the field of optical imaging to be employed as testing mechanisms for various viral pathogens.

### Fluorescence

Fluorescence‐based sensing and imaging offers unique advantages such as good sensitivity, high temporal resolution, availability of biocompatible imaging agents, and noninvasive characteristics that make it relevant in research and clinical settings.^[19]^ Fluorescence‐based optical biosensors are the single largest group of sensors at present, owing to the commercial availability of numerous fluorescent probes, high quality optical fibers and suitable optical instruments.^[20]^ High excitation power is not required for fluorescence, making it a cheap, readily available tool. Wei et al. demonstrated successful imaging of fluorescent nanoparticles and viruses (100 nm and 160–272 nm, respectively) using a setup that fits on a smartphone.^[21]^ While a viable biological imaging technique, there are certain limitations such as fluorophore blinking,^[22]^ photobleaching and orientation of the transition dipoles (causing artifacts), and the inability for many target molecules to exhibit detectable fluorescence signals^[23]^ that need to be overcome before it can be used for virus detection.

Fluorescent biosensors have various parameters like intensity, energy transfer, lifetime and, quantum yield that can be exploited for virus detection.^[24]^ One mechanism that is often used in these biosensors to detect close interactions (<10 nm) between an analyte and a fluorophore is Förster resonance energy transfer (FRET). FRET is the process where incident radiation is absorbed and nonradiatively transferred from a donor to an acceptor by means of long‐range dipole‐dipole coupling.^[25]^ Recent improvements in FRET research and advancements in optical instrumentation have established FRET microscopy as an effective tool for biological imaging and detection applications.^[26]^

The fluorescence emission of sensors can be classified as up‐converting or down‐converting based on their excitation and emission wavelengths. Up‐conversion is when the emitted wavelength is shorter than the excitation wavelength (anti‐Stokes shift). Up‐conversion of the above mentioned NIR 1 excitation wavelengths to shorter visible wavelengths enables minimal autofluorescence, deeper sample penetration depth, higher signal‐to‐noise ratio, and high chemical and physical stability during biosensing.^[27]^ Down‐conversion is the more common mode of linear fluorescence that can exploit NIR 2 by emitting longer wavelengths than the excitation wavelengths.

**Figure 2:**
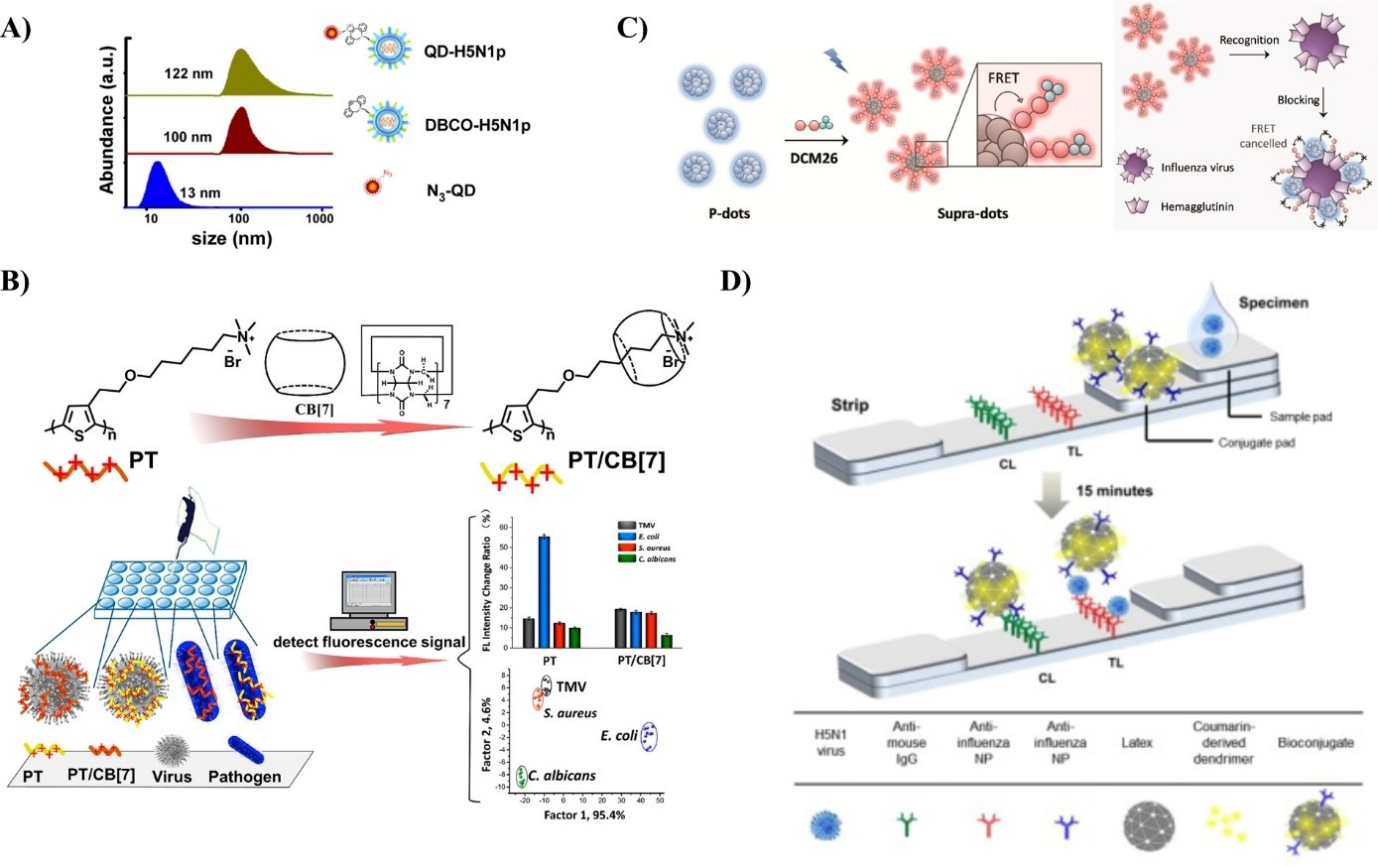
Fluorescence techniques for virus detection. A) Bioorthogonal labeling of H5N1p with NIR QDs for a noninvasive detection method. Reproduced with permission from ref. [19a]; copyright: 2014, American Chemical Society. B) PT and PT/CB[7] synthesis to form a supramolecular structure with TMV and other pathogens resulting in a change in fluorescence intensity. Reproduced with permission from ref. [4]; copyright: 2018, American Chemical Society. C) Formation of supra‐dots from p‐dots and DCM dye molecules causing a decrease in FRET signal when the supra‐dots bind to the hemagglutinin of the influenza virus. Reproduced with permission from ref. [37]; copyright: 2017, American Chemical Society. D) Fluorescence detector flow strip using antibodies to capture antibody‐conjugated latex NPs for the detection of influenza virus. Reproduced with permission from ref. [38]; copyright: 2016, Ivyspring International.

There are multiple different light‐emitting materials (fluorophores) used in fluorescence‐based optical biosensors including inorganic semiconductor quantum dots (QDs), carbon dots (CDs), graphene nanostructures and organic conjugated polymer nanoparticles as shown in Figure 2. QDs, also known as colloidal semiconductor nanocrystals, are small particles (1–10 nm in size in all three dimensions), with unique optical and electronic properties.^[28]^ Due to quantum confinement effects, the emission wavelengths of QDs can be tuned from UV to NIR.^[29]^ Compared to small‐molecule organic dyes, QDs are superior in many aspects, such as high quantum yield, photostability, emission wavelength tunability, Stokes shift, and absorption and emission profiles.^[30]^ They are one of the more commonly investigated materials for fluorescence sensing at present with multiple reports that study their suitability in fluorescent biosensors. For example, their bright photoluminescence, broad size‐tunable emission spectrum, and photochemical stability have been successfully applied for single‐virus tracking *in vitro*.^[19a]^ Pan et al. replaced the native hydrophobic ligands of QDs with multidentate polymer ligands bearing imidazole pendant groups to obtain water‐dispersible azido‐derivatized NIR QDs that were used for tracking and imaging of avian influenza H5N1 pseudotype virus (H5N1p; Figure 2A).^[19a]^ The virus particles were labeled with the water soluble QDs through biorthogonal chemistry, a biocompatible chemical reaction that can occur without interfering with native biochemical processes. This labeling enabled non‐invasive tracking of respiratory viral infection *in vivo*. In another study, a bionic assay was developed for thrombin activity detection (indicator of diseases such as thrombosis, hemophilia, atherosclerosis, and inflammation) based on peptide‐modulated CdTe QD aggregation, where the surface charge of CdTe QDs is regulated by the hydrolysis of a thrombin substrate peptide.^[31]^ However, QDs are mainly made with toxic chemical elements and thus their long‐term toxicity *in vivo* is a major concern.^[30]^ This has led to the development of more biocompatible light‐emitting nanomaterials, such as conjugated polymer nanoparticles (CP NPs) and CDs.^[32]^ However, imaging is more challenging with these materials.^[33]^

Conjugated polymers (CPs) are macromolecules containing backbones with extended π‐conjugated structural units resulting in enhanced light‐harvesting and effective transport of excitons compared to organic dyes. They are an important class of materials due to their signal amplification in therapeutic activities^[34]^ and their reduced photo toxicity by promoting light‐controlled photodynamic therapy.^[35]^ In order to reduce the toxicity of CPs, new water‐soluble CPs have been designed and synthesized, and significant advances in biological applications of these water‐soluble CPs have been made.^[36]^ Bai et al. used the change in fluorescence intensity observed when polythiophene/cucurbit[7]uril (PT/CB[7]) CP formed a supramolecular structure with tobacco mosaic virus (TMV) for virus and other pathogen detection (Figure 2B).^[4]^ Change in fluorescence intensity was observed upon formation of supramolecular structure enabling the detection of TMV virus. This strategy has the potential to detect multiple types of viruses and other pathogens by altering the polythiophene backbone.^[39]^ Due to their capability to undergo conformational changes^[40]^ and encapsulate emitters,^[41]^ CPs are suitable for optical biosensors using FRET.^[42]^ Wang et al. synthesized supra‐dots from poly(9,9‐dioctylfluorene (PFO) nanoparticles (p‐dots) and dicyanomethylene‐4*H*‐pyran (DCM) dye molecules (Figure 2C).^[37]^ The supra‐dots bind to the hemagglutinin of influenza virus resulting in a drastic decrease of FRET signal. The size of CP NPs is important in order to be useful in clinical applications as renal particle filtration is highly dependent on particle size. Kidneys quickly filter out particles smaller than 6 nm, whereas particles larger than 8 nm remain in circulation.^[30]^ To increase the size of small CP NPs, a targeting biomolecule can be linked to particles smaller than 6 nm using cleavable bonds. After imaging, the CP NPs can be released from the biomolecule and excreted through urine. Polymer NPs can be used for *ex vivo* virus detection using fluorescence detector flow strips conjugated with antibodies and dendrimer bioconjugated latex (polystyrene conjugated with aliphatic amines) NPs (Figure 2D).^[38]^

### Surface plasmons

Surface plasmons are collective oscillations of the electron cloud at the surface of a metal excited by incident electromagnetic radiation. There are different types of surface plasmons that are associated with different metal structure types. For example, propagating surface plasmon polaritons (SPPs) are supported by metal films while localized surface plasmon resonances (SPRs or LSPRs) are supported by metal NPs. One of the drawbacks of planar surface plasmon sensors based on SPPs is their extended surface area that requires relatively large sample volumes and large numbers of molecular interactions to generate a detectable signal. A logical approach to overcome these limitations is the use of plasmonic NPs, which exhibit a reduced surface area compared to a metallic film.^[43]^ The development of synthetic methods to control the shape of plasmonic NPs with relatively narrow size distribution has been extensively reported. The shape and size specificity of the SPRs of plasmonic NPs results in homogeneous plasmon line widths and near‐field enhancements, which are crucial to reliable single‐NP and single‐molecule plasmon sensors.

**Figure 3:**
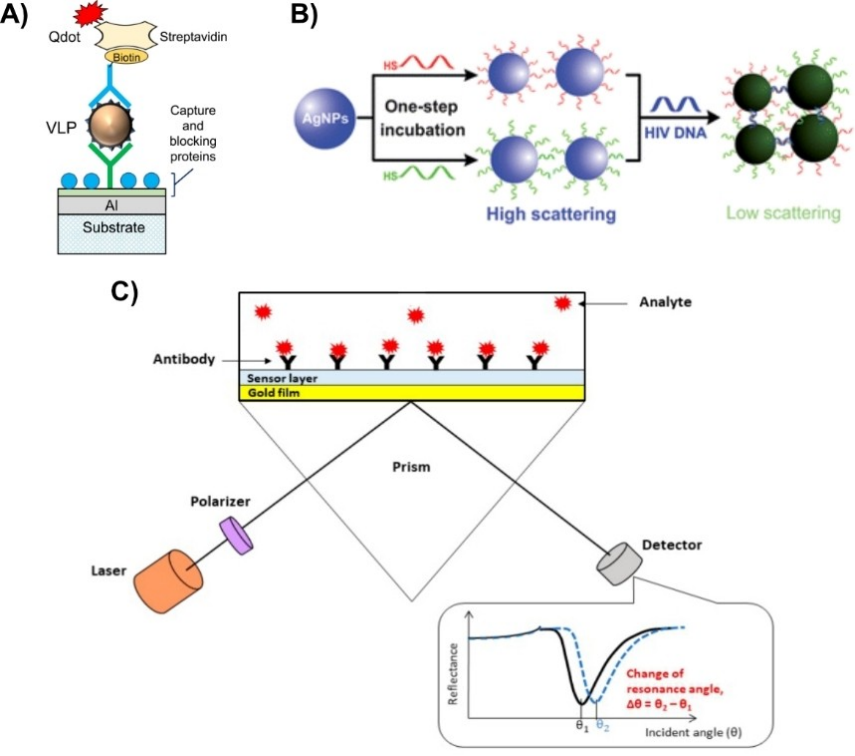
Different SPR techniques for detecting virus particles. A) SPR intensity imaging for norovirus using an antibody‐functionalized plasmonic chip and QD sandwiching technology. Reproduced with permission from ref. [52]; copyright: 2017, Elsevier. B) Scattering‐intensity LSPR detection of hybridized HIV DNA with DNA–AgNPs forming a low‐scattering agglomerate. Reproduced with permission from ref. [53]; copyright: 2012, Royal Society of Chemistry. C) Angle‐dependent Surface plasmon spectroscopy using antibody modified polymer sensor film to bind virus proteins. Reproduced with permission from ref. [54]; copyright: 2020, MDPI.

SPR detection can be modulated on the basis of changes in intensity,^[44]^ refractive index,^[45]^ wavelength,^[46]^ and resonance angle,^[47]^ as shown in Figure 3. SPR is sensitive to phase changes which increases the sensitivity and resolution of SPR detection as compared to conventional methods involving angular and wavelength modulations.^[48]^ The SPR intensity and wavelength can be a function of shape, size, and dielectric constant of plasmonic NPs as well as of the surrounding environment making it an effective tool that can be tailored to imaging and detection of single biomolecules and cells.^[49]^ In 1983, Liedberg et al. created the first plasmonic biosensor by depositing a silver film on glass.^[50]^ Upon adsorption of human γ‐globulin, a shift in the resonance angle of the SPR was observed due to the change in refractive index. The concept of conjugating antibodies with metals has resulted in developing modified DNA‐AuNP aggregates into biosensors that enable detection of nucleic acids of several pathogens.^[51]^ Intensity‐based SPR imaging is the more common commercially available type,^[48]^ employed by companies such as Biosensing Instrument Inc. (http://biosensingusa.com) and Carterra (https://carterra‐bio.com), which detects antibodies, single cells, viruses, proteins, and drug molecules. Ashiba et al. employed a dual antibody trapping system to detect norovirus by adding antibody functionalized QDs to a biosensor modified with virus antibodies.^[52]^ Surface plasmons on an Al film were used to detect the QD intensity as virus particles became trapped between the biosensor antibody and the QD antibody (Figure 3A). Liu et al. took advantage of silver's strong localized SPR light‐scattering signals to prepare thiol‐linked HIV DNA AgNPs.^[53]^ A change in scattering intensity was observed with DNA‐AgNPs hybridized with HIV DNA (Figure 3B). Changes in resonance angle of surface plasmons was utilized by preparing an antibody modified polymer sensor film coated on a gold film that binds dengue virus‐E protein at low detection limits (Figure 3C).^[54]^ The biosensor exhibited an increased shift in the resonance angle upon binding of the virus, due to a change in the refractive index of the environment surrounding the Au film. SPR sensors can provide real‐time 2D resolution of high‐throughput micro‐arrays by combining spectra and phase‐shift investigations.^[48]^ The challenges for SPR detection involve the requirement of high excitation power, toxicity (except gold), and high fabrication costs.

### Plasmon‐enhanced fluorescence

Plasmon‐enhanced fluorescence (PEF) was first investigated in the 1970s by Weber et al. and Knoll et al. using high‐quantum‐yield dye molecules.^[55]^ PEF has been extensively investigated in the context of bioimaging by coupling surface plasmons to periodic corrugations like grating. This coupling yielded enhancement of fluorescent signals over three orders of magnitude.^[56]^ For biological samples Koh et al. reported an enhancement of fluorescence by 20‐ to 30‐fold in the NIR 1 window using a nanostructured plasmonic gold chip.^[57]^ This enables improved detection and bioimaging by fluorescence of biocompatible fluorophores that exhibit relatively low quantum yields.^[58]^ PEF has been frequently used on microfluidic chips for detection and imaging of biomolecules.[^56a^, ^59^] Additionally, PEF is versatile in terms of detecting various types of viruses while improving the detection limits.^[60]^

### Surface‐enhancing Raman scattering

The discovery of the SERS effect in the 1970s demonstrated that Raman scattering efficiency can be enhanced by factors of up to 10^6^ when the sample is located on or near nano‐textured surfaces of plasmonic metals.^[61]^ This enhanced Raman scattering efficiency holds great promise as an optical bioimaging technique, which under optimal conditions, enables deep and high‐resolution volumetric imaging of biological tissues.^[62]^ Solid substrates covered with metal‐coated nanostructures were developed and proposed as efficient and reproducible SERS‐active media.^[63]^ SERS offers higher sensitivities and chemical specificities than most modes of optical detection;^[23]^ and has been used to detect influenza, Adeno, West Nile, and rift valley fever virus amongst others.^[64]^ However, SERS‐based bioimaging is underdeveloped^[64a]^ due to the lack of specialized Raman instruments tailor‐made for volumetric bioimaging,^[65]^ toxicity challenges, and poor stability of SERS probes due to enzymatic degradation or desorption.^[66]^

### Colorimetry

**Figure 4:**
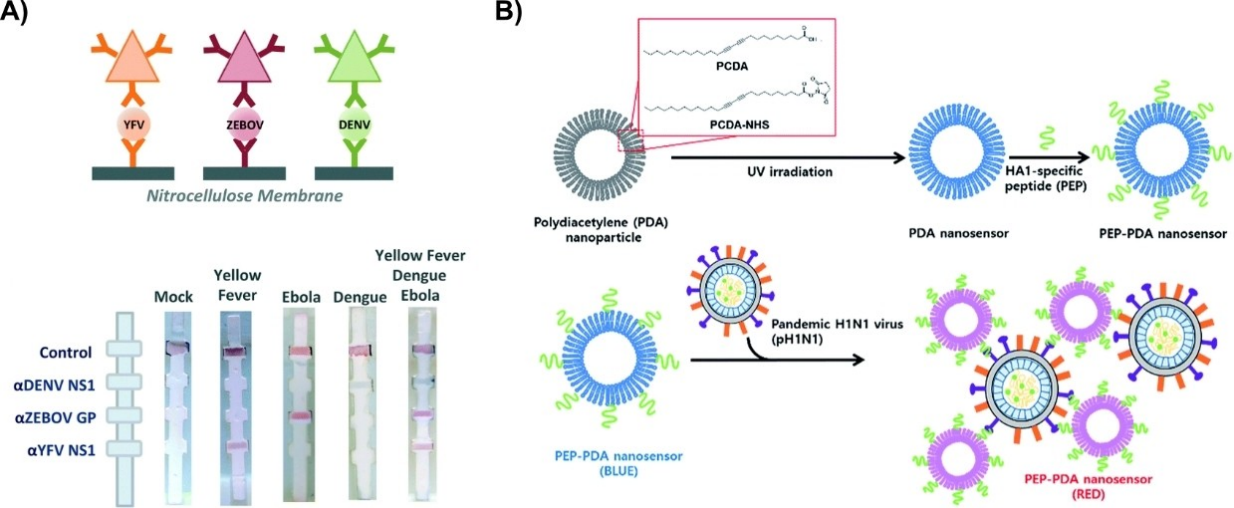
Use of colorimetry in virus detection. A) Detection of dengue (green), yellow fever (orange), and Ebola (red) viruses by binding virus particles between surface conjugated antibodies on a flow device and multicolored antibody conjugated Au nanoplates (depicted by triangles). Reproduced with permission from ref. [71]; copyright: 2015, Elsevier. B) Colorimetric detection of H1 N1 using peptide modified PDA as a nanosensor. Reproduced with permission from ref. [72]; copyright: 2016, Royal Society of Chemistry.

Colorimetric biosensors can detect the presence of particular compounds through a color change easily observable with the naked eye or by a simple optical detector.^[67]^ Due to their ease of use and the fact that they do not require expensive analysis instrumentation, colorimetric biosensors are ideal candidates for POC diagnostics.^[68]^ Smart materials causing visible color change have been developed using noble metal NPs, metal oxide NPs, carbon nanotubes, and CPs,[^40b^, ^69^] as shown in Figure 4. Metal oxide NPs (such as Fe_3_O_4_ and CeO_2_) and carbon nanotubes can produce a color change due to catalyzing the reaction of a peroxidase substrate or their intrinsic peroxidase activity, respectively.[^69b^, ^69c^, ^69d^, ^70^] Several CPs (i. e., polydiacetylene and polythiophenes) can also exhibit a color change that arises from conformational transitions or agglomeration.^[73]^ AuNP are another candidate material for colorimetric biosensors because their color can be manipulated by particles being in an aggregated or non‐aggregated orientation.^[74]^ Extensive research has been performed to modify AuNPs by incorporating various functional acceptors onto their surfaces.^[75]^ However, a major problem with AuNPs is their intrinsic desire to aggregate when in high ionic strength environments or in the presence of impurities.^[76]^ By balancing the interparticle attractive and repulsive forces, and colloidal stability, aggregation can be more easily controlled.^[77]^ Yen et al. used multicolored Au nanoplates (triangular in shape) conjugated with virus specific antibodies to distinguish between dengue, yellow fever, and Ebola viruses.^[71]^ A sandwich hybridization formed between the antibody‐Au nanoplate, virus particle, and a surface‐adhered virus‐specific antibody (Figure 4A). In 1993, Charych et al. proposed that human influenza virus (H1N1) can be detected colorimetrically at lower detection limits by using a polydiacetylene (PDA) bilayer.^[78]^ Since then, PDA bilayers have undergone several modifications in an effort to create a POC testing method.^[79]^ Song et al. introduced a peptide functionalization of PDA that increased the colorimetric detection limit to 10^5^ PFU or 0.2 HAU, similar to the rapid antigen test kits (Figure 4B).^[72]^

## Virus Detection with Optical Detection Techniques

**Figure 5:**
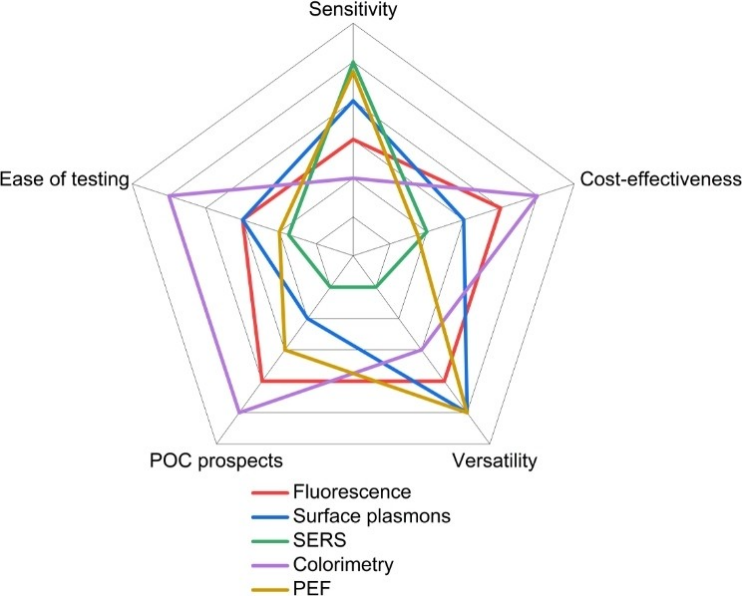
Radar chart comparing fluorescence, SPR, SERS, colorimetry and plasmon‐enhanced fluorescence optical detection techniques as tools in testing different viral pathogens. Different parameters are used to qualitatively compare each technique, including: sensitivity[^71^, ^80^] (detection limit/viral load); cost (instrumentation, fabrication and personnel); versatility^[81]^ (ability to test different pathogens through test modifications); POC prospects;^[82]^ and ease of testing (including testing rate). The further from the center, the higher the relative score the technique received for a particular parameter. This ranking is not absolute and is only provided for the context of this manuscript between the optical phenomena that are discussed.

Each optical detection technique outlined above presents its own advantages and disadvantages, and all have been previously used for virus detection (Figure 5, Table 1). Optical nano‐biosensors dramatically improve the possibilities to monitor *in vivo* processes that enhance our understanding of cellular functions.^[83]^ NPs, QDs, and CPs are the most common types of imaging probes that overcome the limitations of organic dyes.^[30]^ Their ability to undergo surface modifications to increase their hydrophilic or hydrophobic nature is crucial for their use in *in vivo* measurements.^[84]^

**Table 1:** Different optical techniques used to detect various viruses.

Method	Virus	Detection element	Detection limit	Ref.
Fluorescence	Dengue	Immunofluorescence‐assay detecting sandwich complex of antibody conjugated silica microbead and fluorescently labeled dual antibody	1×10^4^ PFU/mL	[80c]
	HIV or hepatitis B	Microbeads optically barcoded by CdSeS QDs coated with capture DNA adhered to a chip	1×10^3^ copies/mL	[91]
	Avian influenza H9N2	Antibody conjugated fluorescent nano‐bioprobes coupled with antibody conjugated immunomagnetic beads to create fluorescent‐biotargeting bifunctional cells	8.94×10^6^ fg/mL	[92]
	Influenza (H1N1 DNA)	Sandwich complex between a CdTe QD and a protein binding aptamer further amplified with streptavidin	3.45×10^6^ fM	[93]
Plasmon‐ enhanced fluorescence	Ebola	Hybrid microfluid and optofluidic device with target RNA functionalized magnetic microbeads	0.2 PFU/mL	[80b]
	Avian influenza H5N1 (rHA protein)	Influenza aptamers immobilized on AgNPs forming a complex with thiazole orange in the presence of rHA protein	3.5×10^6^ fg/mL	[94]
	Influenza	Conjugation of fluorescent QDs, AuNPs, and virus antibodies to peptide linker	17.02 fg/mL	[86]
	Influenza (H1N1 and H3N2)	Binding of antibody conjugated AuNPs and CdSeTeS QDs to virus particles	H1N1: 30 fg/mL (water), 4×10^2^ fg/mL (human serum) H3N2: 10 PFU/mL	[95]
	Norovirus	Norovirus antibody attached to a biosensor chip with QD antigens	1×10^4^ fg/mL (4.3×10^5^ copies/mL)	[52]
	SARS‐CoV	Fluorescently labeled antibodies attached to AuNPs	1×10^2^ fg/mL	[96]
SPR	Ebola	Antiviral immunoglobulins attached to a protein surface coating on a gold layer nanoplasmonic sensor	1×10^5^ PFU/mL	[97]
	Dengue	Surface activation of antigens on a gold chip to attract and covalently couple virus antibodies	1 uL sample	[98]
	Dengue	Antibody modified polymer sensor film to bind virus proteins	8×10^4^ fM	[54]
	HIV DNA	DNA conjugated AgNPs to sandwich HIV DNA forming an agglomerate	1.95×10^5^ fM	[53]
	Hepatitis B (surface antibody)	Hepatitis B surface antibody imprinted on polymer film on a SPR sensor	208.2 mIU/mL	[99]
	Hepatitis B (surface antigen)	Hepatitis B surface antigen bound to SPR sensor	7.81 fg/mL	[80a]
	Avian influenza H5N1	Biotinylated aptamers immobilized on a streptavidin modified gold surface	0.128 HAU	[100]
SERS	Hepatitis B (DNA representative)	DNA‐capture strand coupled to DNA‐reporter strand labeled with a Raman reporter on free AuNPs	0.44 fM	[80d]
	Rift Valley fever virus (RFSV)	Raman reporter coated AuNPs sandwich the virus with antibody conjugated para magnetic NPs	5 fg/mL	[64b]
	Respiratory syncytial	Citrate capped AgNPs that aggregate with an RSV‐antibody sandwich complex	50 fg/mL	[88]
Colorimetry	Dengue, yellow fever, Ebola	Sandwich hybridization between multicolored antibody‐AuNPs, virus particle, and surface adhered antibodies on a flow device	1.5×10^8^ fg/mL (all)	[71]
	Hepatitis B and C	Chip with DNA hybridized AuNPs, enhanced with silver staining	Hepatitis B: 3.6×10^4^ fM; Hepatitis C: 3.6×10^5^ fM	[101]
	Influenza A (H3N2)	Color change induced by antibody conjugated AuNPs attaching to virus receptor probes	7.8 HAU	[102]
	Influenza	Antibody conjugated AuNPs and biotinylated aptamer binding with virus particles on a Dual recognition element LFA	2×10^6^ copies/mL	[103]
	Avian influenza (H5N3, H7N1, H9sN2)	Lateral flow immunoassay with latex particles conjugated with influenza antibody and surface adhered influenza antibodies	H5N3: 6.25×10^3^ PFU/mL; H7N1: 5.34×10^2^ PFU/mL; H9N2: 1.37×10^1^ PFU/mL	[38]
	Zika	Amplified nucleic acids detected with leuco crystal violet on a microfluidic chip	5 PFU/mL	[104]

**Figure 6:**
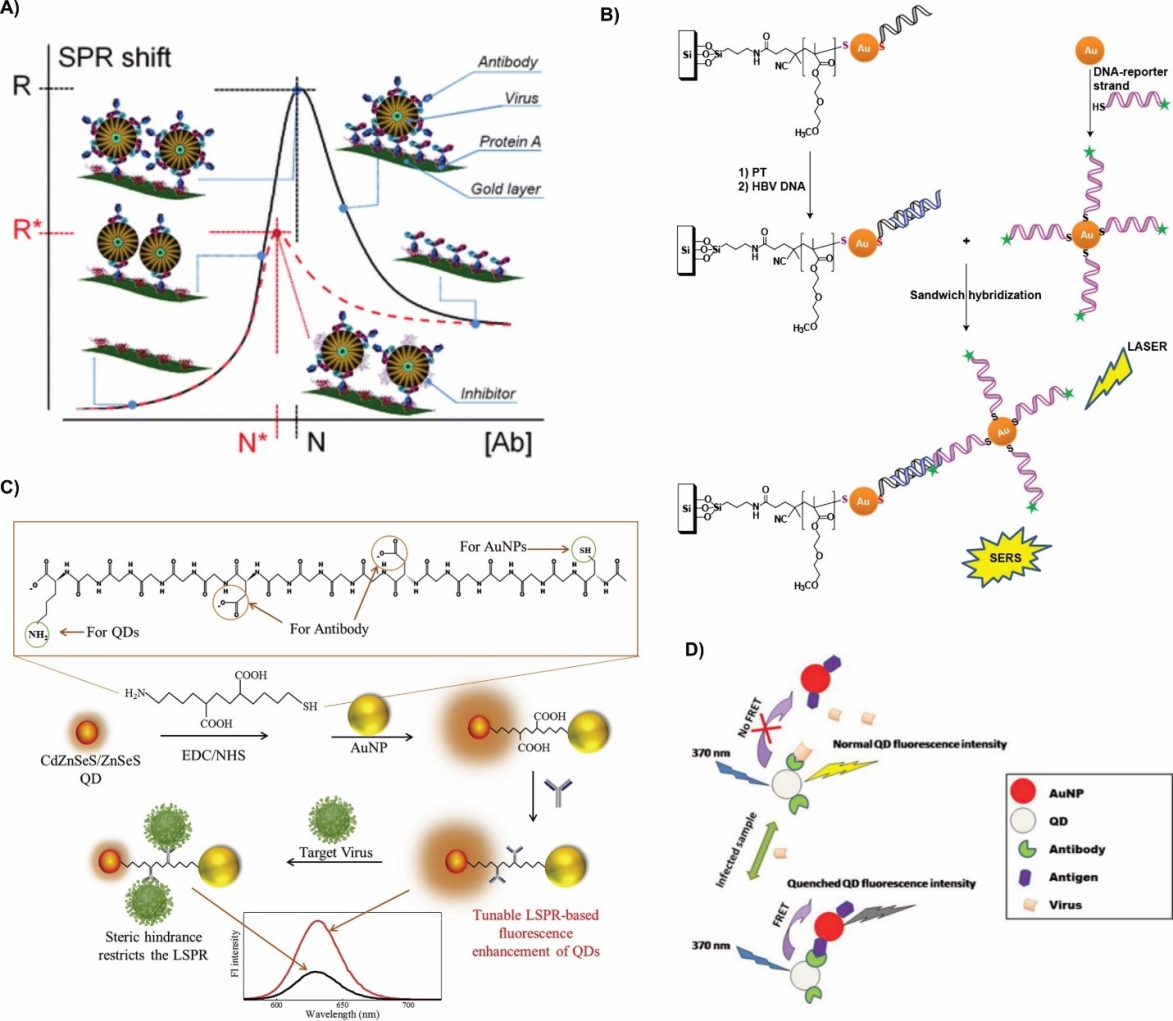
Use of biomolecules for virus detection. A) Inhibition effect of GXM on TMV infection efficiency through wavelength shift of surface plasmons induced by environmental effects. Reproduced with permission from ref. [85]; copyright: 2013, Royal Society of Chemistry. B) SERS imaging using a sandwich hybridization technique to bind hepatitis B DNA (blue) to a DNA‐capture strand (black) and a DNA‐reporter strand (pink) labeled with a Raman reporter (green). Reproduced with permission from ref. [80d]; copyright: 2017, Wiley. C) QD and AuNP peptide conjugate system for the detection of influenza virus by a decrease in the intensity of LSPR. Reproduced with permission from ref. [86]; copyright: 2020, Elsevier. D) Detection of CTV using antibody conjugated CdTe QDs and antigen conjugated AuNPs. Reproduced with permission from ref. [87]; copyright: 2016, Elsevier.

One common approach of optical virus detection is using biomolecules (antibodies, aptamers, DNA) unique to a specific virus as shown in Figure 6. Although this technique has a high selectivity and can be used for a variety of detection modalities, the biomolecules need to be readily available and affordable to be a cost‐effective technique. Boltovets et al. investigated the quantification of the inhibition effect of polysaccharide glucuronoxylomannan (GXM) on the infection efficiency of TMV.^[85]^ This study demonstrated a shift in the wavelength of surface plasmons due to the change in refractive index of the environment induced by the presence of TMV (Figure 6A). Another method by Zengin et al. used SERS to detect DNA representative of hepatitis B on a temperature responsive silicon chip.^[80d]^ AuNPs were modified with a hepatitis B DNA‐capture probe and were adhered to the surface of a chip. DNA reporter strands labeled with indocyanine green were coupled to a different set of AuNPs (Figure 6B). The two sets of AuNPs sandwich hepatitis B DNA making the sandwich complex optically active. The chip was able to be regenerated for multiple uses by a simple sulfate solution washing step. Zhan et al. proposed employing SERS‐based imaging in combination with enzyme immunoassay for the detection of respiratory syncytial virus (RSV).^[88]^ Firstly, a peroxidase solid substrate was modified with 3,3′,5,5′‐tetramethylbenzidine (TMB), a Raman molecule. RSV was captured with a specific antibody on a solid substrate and subsequently bound by another horseradish peroxidase (HRP) labeled antibody to form a sandwich complex. AgNPs were capped with citrate that makes them negatively charged. These negatively charged AgNPs form aggregates upon coming in contact with the positively charged TMB resulting in an enhancement of Raman signal that originates from TMB. LSPR was used by Nasrin et al. who developed a peptide linker conjugated with both a QD and a AuNP which altered the intensity of absorption when influenza virus particles were present (Figure 6C).^[86]^ Shojae et al. have conjugated CdTe QDs with antibodies specific to Citrus tristeza virus (CTV) and AuNPs with the antigen coat protein of CTV.^[87]^ The fluorescence signal from the antibody conjugated QDs was significantly reduced due to FRET from QDs to the AuNPs. For the infected samples, there was a significant increase in the fluorescence signal from the QDs due to the absence of FRET, which resulted in optical detection of CTV.

**Figure 7:**
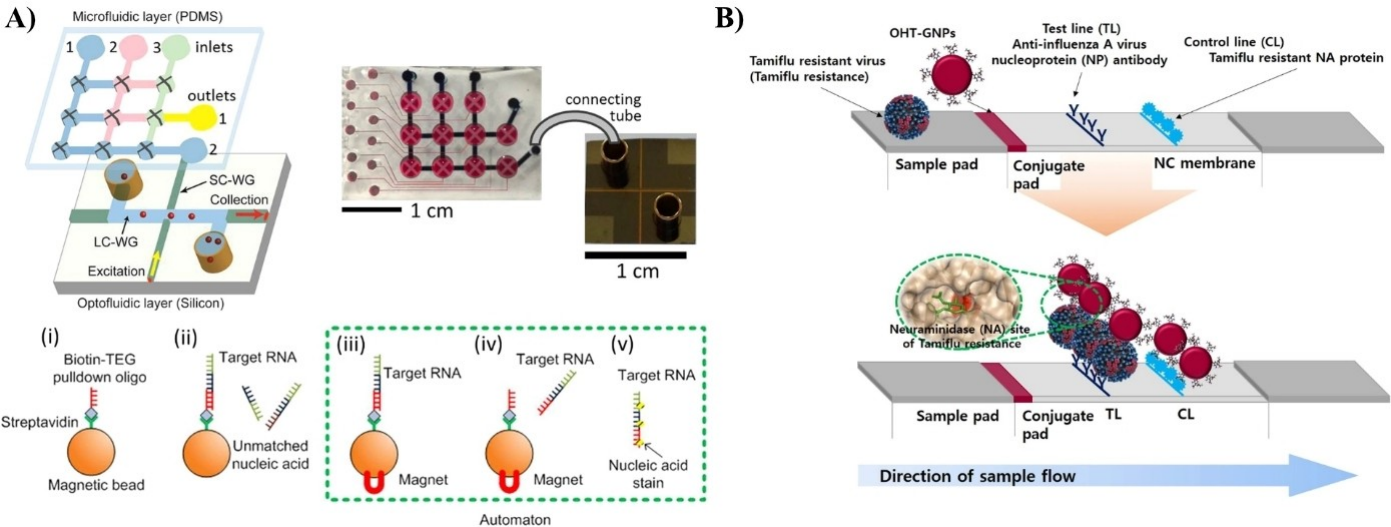
Microfluidic approaches to virus detection. A) A dual fluidic analysis system first uses microfluidics to specifically bind RNA Ebola particles to magnetic oligonucleotide microbeads, then virus RNA chains are thermally released and fluorescently labeled as they are pumped to an optofluidic device for fluorescence‐enhanced SPR detection. Reproduced with permission from ref. [80b]. Copyright: 2015, Nature Research. B) LFA for the detection of TRV by using OHT‐AuNPs that selectively bind to TRV particles and cause a color change when the conjugated system binds to the test line. Reproduced with permission from ref. [90]. Copyright: 2018, Nature Research.

Microfluidics‐based LOC devices have been identified as lead candidates for in‐field POC diagnostics due to their ease of use and low‐cost analysis^[89]^ as shown in Figure 7. Fluorescence enhanced SPR has been shown to detect Ebola virus using a chip prepared with both microfluidic and optofluidic capabilities (Figure 7A).^[80b]^ Oligonucleotide functionalized magnetic microbeads were used to target Ebola RNA for signal amplification, followed by fluorescent labeling and detection, which occurs in under ten minutes. The high specificity, low limit of detection, quick analysis time, and ability to perform multiple runs using a single chip yields a highly desirable method of analysis. Hwang et al. designed a lateral flow assay (LFA) capable of detecting Tamiflu‐resistant influenza virus by using oseltamivir hexylthiol (OHT) AuNPs that selectively bind to Tamiflu resistant virus (TRV).^[90]^ A detection and a control line were prepared with anti‐influenza A virus nucleoprotein antibody and Tamiflu resistant neuraminidase protein respectively (Figure 7B). OHT‐AuNPs bind to TRV particles which bind to the test line antibodies causing a color change due to the conjugation of the AuNPs. The proposed LFA does not require sample preparation and produces results within 10 minutes.

Nano‐biosensors are especially useful as site specific imaging tools to detect where virus particles reside, which is useful for assisting with therapeutic delivery tactics. Recently, Ueki et al. published the first protocol for *in vivo* analysis of lung virus infiltration using a multicolor two‐photon imaging device for influenza virus in mice.^[9b]^ They focused on intravenously administering fluorochrome‐conjugated antibodies to visualize the virus particles in the lungs using two‐photon excitation laser microscopy. This technique allows for viral pathogenicity studies useful for understanding host response mechanisms. Despite their success, the protocol for this technique is laborious and faces several challenges including the lungs constant movement and a limited observation depth of ∼70 μm which is not deep enough for bronchial imaging. An alternative method for single virus tracking uses coherent brightfield (COBRI) microscopy in tandem with fluorescence to track vaccinia virus particles starting before the virus lands on the cell.^[9a]^ A scattering approach is used by shining a continuous wave laser beam at a single wavelength at an aqueous solution containing cells and virus particles and collecting both COBRI and fluorescent images. Both imaging modalities were important because fluorescence confirmed the location of the virus particles as seen on COBRI images. COBRI images could then be used to estimate the *x*,*y*,*z* planar positions to determine when a single virus particle is interacting with a cell surface. They were able to successfully capture a single virus particle diffusing in the aqueous solution and landing on the cell plasma membrane. This work is extremely useful for studying the delivery of nanoparticles or therapeutics into cells. Studying viruses in their physiological environments, provides insight into host response mechanisms assisting in developing more direct therapeutic targeting.

## Implementation of Optical Techniques for SARS‐CoV‐2 Detection

The race to design a novel biosensor that would optimize COVID‐19 tests in terms of cost, rate of testing and sensitivity is underway. Although imaging‐based biosensing techniques are more expensive and complicated as a method for virus detection, they are useful in providing insight into better understanding the virus replication pathway and can help in developing treatment options. Biosensors have a unique capability for surface modification to create targeting sensors through attachment of biomolecules that detect specific viral sequences. SARS‐CoV‐2’s DNA‐RNA hybridization is used in RT‐PCR amplification and can be induced by thermal exposure at a temperature slightly lower than the nucleic acid strand melting temperature.^[105]^ Qiu et al. used this phenomenon to develop a dual‐functional gold nanoisland‐based biosensor using both thermoplasmonic heating (to induce signal amplification) and SPR imaging to detect RNA‐dependent RNA polymerase (RdRp), a closely related nucleic acid sequence of SARS‐CoV‐2, at a 0.22 pM detection limit.^[12a]^ At this concentration, a 200 μL analyte solution contained ∼2.26×10^7^ copies of the RdRp‐COVID sequence, which is higher than the viral load of SARS‐CoV‐2 virus soon after onset (1×10^6^ copies/mL).^[106]^ The biosensor used a thiol‐cDNA functionalized gold surface that immobilizes RdRp when heated at 41 °C. Although this biosensor can quickly detect the presence of RdRp at a low detection limit, it is a single‐use apparatus and requires sample preparation prior to analysis.

Several LOC diagnostic systems have received emergency use authorization (EUA) from the U.S. Food and Drug Administration (FDA).^[11]^ Abbott™ (www.abbott.com), BioFire™ (www.biofiredx.com), and Cepheid™ (www.cepheid.com) use nucleic acid amplification followed by fluorescent labeling to detect the presence of SARS‐CoV‐2 quickly with more accurate results. CapitalBio™ (www.capitalbiotech.com) uses similar diagnostics to screen for six common respiratory viruses; however, it requires 1.5 hours to generate results. Canon™ (https://global.medical.canon) uses loop‐mediated isothermal amplification (LAMP), a similar nucleic acid amplification technique to PCR,[^107^, ^108^, ^109^] to generate results in 40 minutes with 100 % specificity and >90 % sensitivity. These companies have produced nucleic acid amplification instruments that are portable and do not require trained operators. Several research labs have confirmed the use of LAMP for nucleic acid amplification, which causes a color change upon addition of colorimetric indicators when enough virus DNA units are present, thereby removing the need for fluorescent labeling and detection, as an easy platform for SARS‐CoV‐2 virus detection.[^108b^, ^110^]

A lateral flow immunoassay (LFIA) was developed by Chen et al. to test for the presence of anti‐SARS‐CoV‐2 Immunoglobin G (IgG) antibodies in human serum.^[111]^ Lanthanide‐doped polystyrene nanoparticles (L‐NPs) that serve as fluorescence reporters were prepared and functionalized with either mouse anti‐human IgG (M‐HIgG) or rabbit IgG (rIgG) antibodies. A test line was coated with recombinant SARS‐CoV‐2 nucleocapsid phosphoprotein and a control line with goat anti‐rabbit IgG. As human serum was introduced onto the flow assay, human anti‐SARS‐CoV‐2 IgG antibodies conjugated with M‐HIgG@L‐NPs and attached to the test line material causing a change in the fluorescent signal at the test line. Similarly, rIgG@L‐NPs conjugated with the control line material. The ratio of the test line to control line fluorescence signal was calculated to determine the concentration of anti‐SARS‐CoV‐2 IgG in the human serum sample. A similar method was developed by Li et al. who could identify the presence of immunoglobulin M (IgM), the first line of defense against viral infections, and IgG, long‐term immunity antibodies, in as little as 15 minutes from unprocessed blood samples.^[112]^ SARS infection cases reported the presence of IgG antibody in patients’ blood after 5 days and IgM after 10 days.^[113]^ Due to SARS‐CoV‐2’s similar viral make‐up,^[114]^ it is hypothesized that similar detection methods can be achieved for COVID‐19 patients. These methods offer an affordable, user friendly, fast test for the presence of anti‐human SARS‐CoV‐2 antibodies; however, the delayed emergence of antibodies could make these methods non‐viable for recently infected patients.

Of particular interest is the biophysical property of the SARS‐CoV‐2 virus to recognize and bind to angiotensin‐converting enzyme 2 (ACE‐2) at a high affinity.^[115]^ A LFIA can be designed with AuNPs conjugated with SARS‐CoV‐2 spike antibody, which has shown promise as a field‐effect transistor detection method for SARS‐CoV‐2,^[12b]^ to bind to SARS‐CoV‐2 virus particles. By modifying the test line with recombinant ACE‐2 protein, a simple, no‐sample‐preparation colorimetric method could be prepared to detect for SARS‐CoV‐2. While this set‐up might not be the most cost‐effective method due to the high price of ACE‐2 recombinant protein (50 μg/$400; www.acrobiosystems.com) and the limited supply and high cost of SARS‐CoV‐2 spike antibodies (100 μg/$375; www.genscript.com), it has the potential to be used as an at home test for SARS‐CoV‐2. Similar strategies using fluorescence can be implemented using the previously described sandwich method by trapping the virus particle between a similar antibody conjugated NP and recombinant ACE‐2 protein attached to a surface. By designing the NPs using either gold or other SPR‐enhanced technology, the amount of sample necessary to provide a positive readout can be minimized. This reduces the amount of recombinant ACE‐2 protein and SARS‐CoV‐2 spike antibodies compared to the amount necessary to cause a colorimetric change, thereby minimizing cost. Ideally, this system can be designed with a washing step to remove the bound virus particles to prepare a reusable sensor.^[116]^ Combining LOC or microfluidic technology that first purifies and removes other proteins before introducing the remaining sample to the sensor has the ability to further increase the selectivity and reduce the cost and cleaning required for multiple sample uses.

There has been extensive research on optical detection of virus particles by sandwiching them between either primary and secondary antibodies or between a peptide sequence and an antibody. Recent studies by Wu et al. and Chen et al. have provided evidence for antibodies that specifically bind to SARS‐CoV‐2.^[117]^ These studies can further enhance the synthesis of optical biomarkers that can be successfully conjugated with the antibodies of SARS‐CoV‐2. Advanced therapeutic studies have been carried out for targeting the spike protein in SARS‐CoV‐2 that binds to ACE‐2 receptor in host cells.^[118]^ The findings of these studies can be employed to design and synthesize aptamers that specifically bind to SARS‐CoV‐2. Designing and fabricating optical biosensors based on the receptors of SARS‐CoV‐2 could be an effective method for COVID‐19 testing.

## Conclusions and Future Prospective

COVID‐19 has had a dramatic effect on the world, causing disruptive stay‐at‐home orders and an unprecedented surge in hospital demand for both treatment and diagnosis. Science communities have been tasked with developing new methods for detection and therapeutics for SARS‐CoV‐2. Facilities with RT‐PCR equipment have been inundated with samples for analysis; however, lack of sufficient RT‐PCR equipment and experienced personnel limit the number of samples that can be run. This has resulted in RT‐PCR being a major bottle‐neck for COVID‐19 testing. There is a growing need for novel biosensors that can match the sensitivity and selectivity of conventional nucleic acid tests. Nanomaterial‐based biosensors present ideal alternatives due their advancement in design and fabrication methods, high selectivity, low sensitivity, and good reproducibility. Any biosensor used for COVID‐19 and future viral outbreaks should be readily accessible, affordable, and ideally used either as an at‐home test or with minimal sample preparation. Optical biosensors provide additional and alternate methods of testing that have the potential to generate results at a much quicker rate than the tests that are currently being used for COVID‐19 testing. This minireview highlighted the current trends in optical biosensors and their ability for virus detection. Novel viral outbreaks will occur in the future, and it is necessary to have virus sensing technology in place to help aid in detection and to reduce virus spread.

## Conflict of interest

The authors declare no conflict of interest.

## Biographical Information

*Hemanth Maddali received his integrated masters degree from the University of Hyderabad in 2015. During his Masters, he worked on organic photonic micro‐resonators in Prof. Chandrasekar Rajadurai's lab. He then moved to NCBS, Bengaluru for an internship, where he worked with Dr. Sunil Laxman on mass spectrometry‐based analysis of metabolomics. He then moved to Rutgers to pursue his PhD in chemistry under the supervision of Prof. Deirdre O'Carroll with research focusing on doped conjugated polymer thin films for various optoelectronic devices*.

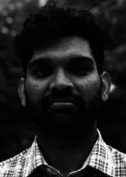

## Biographical Information

*Catherine E. Miles received her bachelor's degree in chemistry from Western Washington University in 2016 where she worked in the lab of Prof. Mark Bussell focusing on developing catalysts for the removal of sulfur and nitrogen impurities from crude feed oil. She then moved to Rutgers University to pursue her PhD in chemistry under the guidance of Prof. Joachim Kohn, where she studies the effect of structure‐property relationships on drug delivery and degradation of tyrosine‐derived polymer microparticles*.

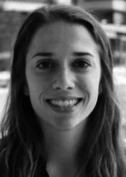

## Biographical Information

*Joachim Kohn, PhD, FBSE leads the Laboratory for Biomaterials Research at Rutgers University. Kohn has made seminal contributions to the design, synthesis, characterization and fabrication of new biomaterials for regenerative medicine, tissue engineering and drug delivery. He pioneered the use of combinatorial and computational methods for the optimization of biomaterials for specific medical applications. Medical devices (a coronary stent and an antimicrobial device to prevent infections in pacemaker patients) are based on polymers developed by Kohn and have been implanted in about 500 000 patients in 46 countries*.

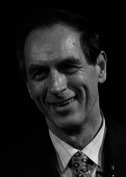

## Biographical Information

*Deirdre M. O′Carroll is an Associate Professor in the Departments of Materials Science & Engineering and Chemistry & Chemical Biology at Rutgers University. Prior to joining Rutgers, she conducted postdoctoral research in plasmonics at California Institute of Technology in the US and at the University of Strasbourg and CNRS in France. She is a recipient of a National Science Foundation CAREER Award (2016) and an American Chemical Society Young Investigator Award (2017). Her research areas include nanophotonics and polymer semiconductors*.